# Diagnostic Utility of Vestibular Markers in Identifying Mild Cognitive Impairment and Early Alzheimer’s Disease in Older Adults

**DOI:** 10.3390/jcm14134544

**Published:** 2025-06-26

**Authors:** Khalid A. Alahmari, Sarah Alshehri

**Affiliations:** 1 Physical Therapy Program, Department of Medical Rehabilitation Sciences, College of Medical Applied Sciences, King Khalid University, Abha 61421, Saudi Arabia; kahmarie@kku.edu.sa; 2 Otolaryngology, Head and Neck Surgery, Department of Surgery, College of Medicine, King Khalid University, Abha 61421, Saudi Arabia

**Keywords:** cognitive impairment, vestibular function, vHIT, posturography, Alzheimer’s disease

## Abstract

**Background/Objectives**: Cognitive impairment and vestibular dysfunction commonly co-occur in older adults and may share overlapping neuroanatomical pathways. Understanding their association may enhance the early identification of cognitive decline using clinically feasible vestibular assessments. This study aimed to examine the relationship between vestibular dysfunction and early cognitive impairment, assess the diagnostic accuracy of vestibular markers, and explore the association of subjective dizziness and balance measures with cognitive performance. **Methods**: Our cross-sectional study included 90 participants aged ≥60 years, classified into cognitively healthy, mild cognitive impairment (MCI), and early Alzheimer’s disease (AD) groups. Cognitive function was assessed using the MoCA and the MMSE; vestibular function was evaluated via posturography sway and horizontal vHIT gain. Subjective dizziness and balance were measured using the Dizziness Handicap Inventory (DHI), gait speed, and eyes-closed balance time. The data were analyzed using SPSS v24 with ANOVA, Pearson correlations, linear regression, and ROC curve analyses. **Results**: Significant group differences were found across the cognitive and vestibular scores (MoCA: *p* = 0.001. Sway: *p* = 0.001. vHIT: *p* = 0.001). vHIT gain and posturography sway independently predicted the MoCA and MMSE scores (adjusted R^2^ = 0.68 and 0.65, respectively). The ROC analysis showed a strong diagnostic accuracy for posturography sway (AUC = 0.87) and vHIT gain (AUC = 0.82). **Conclusions**: Vestibular dysfunction is significantly associated with early cognitive impairment and may serve as a useful clinical marker for cognitive screening in older adults.

## 1. Introduction

Aging is associated with a gradual decline in sensory, motor, and cognitive functions, which collectively impact the independence and quality of life of older adults [1]. Among these domains, cognitive impairment—ranging from mild cognitive impairment (MCI) to Alzheimer’s disease (AD)—poses a significant public health burden globally [2]. The early identification of cognitive deficits is critical, as interventions are more effective when initiated during the prodromal stages of neurodegeneration [3]. Parallel to cognitive decline, vestibular dysfunction is increasingly recognized in the aging population, manifesting as impaired balance, dizziness, and an increased risk of falls [4]. Given the shared neuroanatomical substrates between vestibular processing and cognitive regulation—such as the hippocampus, temporoparietal cortex, and cerebellum—emerging evidence suggests a functional relationship between these two domains [5].

Recent research has shown that vestibular impairments are more prevalent among individuals with cognitive deficits than in cognitively intact older adults [6]. Studies have identified structural and functional vestibular alterations in patients with MCI and AD, with findings of reduced vestibulo-ocular reflex (VOR) gain, increased postural sway, and impaired spatial navigation [7]. These deficits are not solely attributable to age-related degeneration, as vestibular decline in cognitively impaired individuals often exceeds that seen in age-matched controls [4]. Anson et al. [8] and Chari et al. [6] demonstrated that vestibular loss was independently associated with lower performance in memory and executive function tasks. However, while these studies provide important insights, few have systematically evaluated both subjective and objective vestibular measures in relation to standardized cognitive assessments across well-defined diagnostic groups in a single study.

There remains a notable gap in the literature regarding the clinical utility of vestibular assessment as a complementary tool in the early detection of cognitive impairment. Most previous work has focused on isolated measures or non-standardized vestibular protocols, limiting its comparability and clinical translation. Furthermore, the diagnostic accuracy of specific vestibular markers, such as posturography sway and vHIT gain, has not been comprehensively quantified in relation to MCI and early AD classification [9]. Additionally, the relationship between functional balance indicators (e.g., gait speed and balance duration) and subjective dizziness symptoms with cognitive performance has not been thoroughly examined using multivariable models controlling for potential confounders, such as age, education, and comorbidities [10]. Given the high prevalence of both vestibular dysfunction and cognitive impairment in older adults, clarifying these associations using clinically applicable tools is essential for advancing early screening strategies [10].

The primary objective of this study was to evaluate the association between vestibular dysfunction—measured objectively using posturography and horizontal video head impulse tests (vHITs)—and early cognitive impairment in older adults. Secondary objectives included assessing the correlation between vestibular markers and cognitive performance scores (the MoCA and the MMSE); determining the diagnostic accuracy of posturography sway and vHIT gain in identifying mild cognitive impairment and early Alzheimer’s disease; and examining the relationship between subjective dizziness, functional balance measures (gait speed and eyes-closed balance time), and patients’ cognitive status. It was hypothesized that vestibular dysfunction would be significantly associated with cognitive impairment and that specific vestibular markers—namely posturography sway and horizontal vHIT gain—would demonstrate a strong diagnostic utility in distinguishing cognitively impaired individuals from cognitively healthy controls.

## 2. Materials and Methods

### 2.1. Design

This cross-sectional observational study was conducted between 12 June 2023 and 13 March 2024 at the vestibular clinic of King Khalid University Medical City, Abha, Saudi Arabia. Ethical approval was obtained from the institutional review board of King Khalid University Medical City (IRB No. ECM#2025-713), and written informed consent was obtained from all the participants prior to enrolment. The study was conducted in full compliance with the ethical principles outlined in the Declaration of Helsinki, and confidentiality and data protection protocols were strictly observed throughout the research process.

### 2.2. Participants

The participants for this study were older adults aged 60 years and above who were referred to the vestibular clinic at King Khalid University Medical City between June 2023 and March 2024 for cognitive and/or balance evaluations. Eligible individuals were screened and categorized into three diagnostic groups: cognitively healthy, MCI, and early AD. The diagnostic classification was established using internationally recognized clinical criteria [11]. The cognitively healthy participants demonstrated normal performance on cognitive screening tools, with no subjective memory complaints or functional impairments. MCI and early AD were diagnosed in the neurology department and referred to the vestibular clinic. MCI was diagnosed based on the Petersen criteria, which include evidence of subjective cognitive complaints, objective impairment in one or more cognitive domains (z-score ≤ −1.5), preserved activities of daily living, and the absence of dementia [12]. Early AD was diagnosed in accordance with the National Institute on Aging–Alzheimer’s Association (NIA-AA)’s core clinical criteria, based solely on clinical evaluations and cognitive test results without neuroimaging or biomarker confirmation, which require evidence of progressive memory decline, impairment in daily functioning, and objective deficits consistent with AD to be identified via cognitive assessments [13].

The inclusion criteria comprised the following: (1) age ≥ 60 years, (2) the ability to provide informed consent, (3) the capacity to participate in cognitive and vestibular assessments, and (4) classification into one of the three diagnostic categories. The exclusion criteria included the following: (1) a history of diagnosed vestibular disorders unrelated to aging or neurodegeneration (Ménière’s disease, vestibular neuritis), (2) neurological conditions other than AD (e.g., Parkinson’s disease, stroke), (3) significant uncorrected hearing or visual impairments, (4) psychiatric illness (e.g., major depression, schizophrenia) that could confound cognitive testing, and (5) the use of medications known to affect vestibular function (vestibular suppressants) within two weeks of assessment. Additionally, the participants with previously confirmed diagnoses of bilateral vestibular loss or presbyvestibulopathy were not identified in the sample. Clinical screening by otology specialists ensured that only age-appropriate vestibular changes were included, and no participant met the diagnostic criteria for these conditions during recruitment. This approach preserved the focus on age-related vestibular–cognitive interactions while minimizing the confounding influence of non-degenerative vestibular disorders.

The participants were recruited consecutively from clinic referrals and underwent an initial eligibility screening followed by structured interviews and assessments conducted by trained clinicians. Their cognitive status was confirmed using the Montreal Cognitive Assessment (MoCA) [14] and the Mini-Mental State Examination (MMSE) [15], while their functional independence was assessed through clinical interviews. The individuals meeting the inclusion criteria were enrolled after providing written informed consent. The final sample included equal representation across the three diagnostic groups, ensuring comparability in age and sex distribution while allowing for the evaluation of vestibular–cognitive relationships.

### 2.3. Variables

This study involved a comprehensive assessment of cognitive function, vestibular performance, subjective dizziness, and functional balance. All the variables were collected through standardized protocols administered by trained clinicians (a consultant physiotherapist specializing in vestibular rehabilitation and an otology consultant) in a controlled clinical setting. The following categories of variables were included:

#### 2.3.1. Cognitive Function

- Montreal Cognitive Assessment (MoCA)

The MoCA was used as a primary cognitive screening tool to evaluate global cognitive function across multiple domains, including memory, visuospatial ability, executive function, attention, language, and orientation [14]. The test was administered in its standard Arabic version and validated for use in elderly Saudi populations. The Arabic version of the MoCA used in this study has been previously validated for older, Arabic-speaking populations, demonstrating acceptable reliability and construct validity for cognitive screening in regional settings [16]. The MoCA yields scores ranging from 0 to 30, with higher scores reflecting better cognitive performance. A score below 26 was used to indicate possible cognitive impairment, in line with the established clinical cutoffs. Similarly, a Mini-Mental State Examination (MMSE) score below 27 was also considered suggestive of cognitive decline. Both assessments were administered by trained personnel in a quiet clinical environment, with each session lasting approximately 10–15 min.

The study focused on using these well-established screening tools to ensure methodological feasibility and consistency within routine clinical settings. As such, comprehensive neuropsychological batteries assessing specific cognitive subdomains were not employed. This approach prioritized practical implementation but limited the ability to analyze domain-specific cognitive impairments. Future studies incorporating detailed neuropsychological assessments may provide a more granular understanding of the vestibular–cognitive relationship.

Although an MoCA cutoff score of <26 was applied as a general screening threshold, the final diagnostic classification for mild cognitive impairment and Alzheimer’s disease was made by neurologists based on internationally accepted clinical criteria. To account for any variation in cognitive performance due to patients’ educational background, years of education were included as a covariate in all the regression models, reducing any potential misclassification and improving the robustness of the cognitive group assignments.

b. Mini-Mental State Examination (MMSE)

The MMSE was used as a complementary tool to the MoCA, providing an additional global cognitive index [15]. It assesses orientation, registration, attention, calculation, recall, language, and visuoconstructional skills. The Arabic version was employed, and the scores also ranged from 0 to 30, with lower scores indicating greater cognitive impairment. The MMSE’s administration adhered to the standardized protocol, requiring approximately 7–10 min per participant.

#### 2.3.2. Vestibular Function

- Posturography Sway Score (cm^2^)

Posturography was conducted using a computerized static force platform system (Technobody, Dalmine, Italy), which provides a high-resolution analysis of postural stability through center-of-pressure (COP) tracking [17,18]. The participants were instructed to stand barefoot on the force platform in a standardized Romberg position, with their feet together, arms relaxed by their sides, and eyes closed to minimize the visual input. Each trial lasted 30 s, during which the system continuously recorded anteroposterior and mediolateral COP displacements at a sampling frequency of 100 Hz. The primary outcome measure was the total sway area, automatically computed by the proprietary software as the 95% confidence ellipse area (cm^2^) encompassing the COP trajectory over the duration of the trial. Three separate trials were performed, with 1 min rest intervals between the trials to reduce fatigue. The participants were monitored throughout for safety, and the trials were repeated if movement artifacts or interruptions were noted. The mean of the three trials was calculated and used for the statistical analysis. Increased sway area values indicated poorer postural control, suggesting compromised vestibular or sensory integration function. This procedure aligns with the standardized methods reported in previous studies of aging and balance assessment [17,19], and the equipment used complies with the ISO standards for clinical balance evaluation.

b. Video Head Impulse Test (vHIT)—Horizontal Vestibulo-Ocular Reflex (VOR) Gain

The video head impulse test (vHIT) was performed using the EyeSeeCam vHIT system (Natus Medical Inc., Pleasanton, CA, USA) [20]. The participants were seated upright in a well-lit room approximately 1.5 m away from a fixed visual target placed at eye level. The test was administered by a trained clinician following standardized procedures for clinical vHIT assessment. Each participant wore a lightweight headband fitted with high-speed cameras (sampling rate \~250 Hz) that continuously recorded eye and head velocities. The examiner applied brief, passive, and unpredictable horizontal head impulses of approximately 10–15° amplitude and \~2–5 Hz frequency, with peak head velocities exceeding 150°/s, replicating the physiological head movements encountered in daily activities. The impulses were delivered in both leftward and rightward directions while the participant maintained a gaze on the target. For each side, a minimum of thirty artifact-free impulses were collected. The system software automatically calculated the horizontal VOR gain by dividing the area under the eye velocity curve by the area under the head velocity curve for each impulse. A mean gain value of <0.80 was considered indicative of vestibular hypofunction, consistent with the established clinical cutoffs [21]. All the testing was completed in one session, with the participants being provided rest as needed to avoid fatigue or test-related discomfort. While vHIT gain was the primary outcome, corrective saccades were visually monitored but not quantitatively analyzed. Adequate fixation on the visual target was ensured through standardized instruction and demonstration before testing. The participants who failed to maintain their gaze or comprehend the task requirements were excluded. Multiple impulses were performed until a minimum of six artifact-free trials were obtained per side. Although fixation compliance was verified in all the included participants, the absence of a formal saccade analysis and the potential variability in target tracking among cognitively impaired individuals is acknowledged as a methodological limitation.

#### 2.3.3. Subjective Vestibular Symptoms

Dizziness Handicap Inventory (DHI)

The DHI is a 25-item, self-reported questionnaire that evaluates the perceived impact of dizziness on daily activities [22]. It consists of three subdomains: functional, emotional, and physical. Each item is scored as “Yes” (4 points), “Sometimes” (2 points), or “No” (0 points), yielding a total score from 0 to 100. Higher scores reflect greater perceived disability [22]. The validated Arabic version was administered verbally to the participants, and their responses were recorded by the investigator.

#### 2.3.4. Functional Balance Performance

- Gait Speed (m/s):

Gait speed was assessed using the 4 m walk test on a flat, unobstructed surface. The participants were instructed to walk at their usual pace, and the timing was initiated when the first foot crossed the start line and stopped when the first foot crossed the 4 m line [23]. The time was measured using a stopwatch, and the average speed (m/s) was calculated. Two trials were performed, and the mean value was recorded. The inter-rater reliability of the gait speed measurements was maintained through the standardized training of the assessors and the use of duplicate timing.

b. Balance Test Time (seconds):

Static balance was measured under eyes-closed, single-task conditions using the Romberg stance [24]. The participants stood barefoot with their feet together and arms at their sides while maintaining balance for up to 30 s. The timing was stopped if the participant moved their feet, opened their eyes, or required support. Three trials were conducted, and the best performance (longest duration) was used for the analysis. All the participants performed the Romberg test with their feet together and their eyes closed for up to 30 s. The trials were separated by 1 min rest intervals to reduce fatigue. The balance time was measured in seconds using a stopwatch, starting once the participant assumed the correct position. A trial was terminated—and the timing stopped—if the participant opened their eyes, moved their feet, raised their arms, or required external support. The longest of three trials was recorded as the final score.

#### 2.3.5. Demographic and Clinical Covariates

- Age, Sex, and Education

Demographic data were recorded through structured interviews. Age was recorded in years, sex was documented as male or female, and years of formal education were reported as continuous variables.

b. Comorbidities

The presence of hypertension and diabetes mellitus was recorded based on physician diagnosis and current medication use, confirmed via medical records.

c. Body Mass Index (BMI)

The BMI was calculated using the standard measurements of weight (kg) and height (m^2^) taken during the clinic visit. The formula BMI = weight (kg)/height (m^2^) was applied.

d. Fall History

The participants were asked about any falls experienced in the past 12 months, with their responses categorized as “Yes” or “No.” A fall was defined as “an unexpected event in which the participant comes to rest on the ground, floor, or lower level”.

### 2.4. Sample Size Estimation

The sample size for this cross-sectional study was determined using G*Power statistical software (version 3.1.9.7) to ensure sufficient power to detect differences in vestibular function across cognitive status groups. An a priori power analysis was conducted for a one-way ANOVA with three groups—cognitively healthy, MCI, and early AD—a medium effect size (f = 0.30) [25], an alpha level of 0.05, and a desired power of 0.80. Based on these parameters, the minimum required sample size was calculated to be 84 participants. To account for potential dropouts or incomplete data, the sample size was increased to 90 participants, with 30 individuals allocated to each group.

### 2.5. Data Analysis

The data were analyzed using IBM SPSS Statistics version 24.0 (IBM Corp., Armonk, NY, USA). Descriptive statistics were calculated for all the demographic, clinical, cognitive, vestibular, and balance-related variables. Continuous variables were expressed as means and standard deviations, while categorical variables were presented as frequencies and percentages. As the data followed a normal distribution, parametric tests were employed throughout the analysis. The normality of the continuous variables was assessed using the Shapiro–Wilk test. As the data met the assumptions for normal distribution, parametric tests were applied. All the statistical tests were two-tailed, and a significance level of α = 0.05 was used throughout. A one-way analysis of variance (ANOVA) was used to compare the cognitive and vestibular function scores, DHI scores, gait speed, and balance time across the three diagnostic groups (cognitively healthy, MCI, and early AD), followed by Tukey’s post-hoc test for pairwise group comparisons. Chi-square tests were used to compare the categorical variables. Pearson correlation coefficients were calculated to examine associations between the cognitive scores and the vestibular parameters. Stepwise multivariable linear regression models were constructed to identify independent predictors of the MoCA and MMSE scores, adjusting for relevant covariates, including age, education, comorbidities, DHI score, gait speed, vHIT gain, and posturography sway. Specifically, the covariates entered into the regression models included age (years), education (years), the presence of hypertension and/or diabetes (binary), the Dizziness Handicap Inventory (DHI) score, gait speed (m/s), horizontal vHIT gain, and the posturography sway score (cm^2^). Variance inflation factors (VIFs) were examined to assess multicollinearity. A receiver operating characteristic (ROC) curve analysis was performed to determine the discriminative ability of the vestibular markers (vHIT gain and posturography sway) in identifying cognitive impairment, with the area under the curve (AUC); 95% confidence intervals; and sensitivity, specificity, and optimal cutoff values being derived using Youden’s Index. A *p*-value < 0.05 was considered statistically significant for all the analyses.

## 3. Results

The participants with early Alzheimer’s disease were significantly older and had fewer years of formal education compared to those with mild cognitive impairment and the cognitively healthy controls, with medium-to-large effect sizes (η^2^ = 0.09 and 0.12, respectively; *p* = 0.014 and *p* = 0.003) (Table 1). Additionally, the body mass index showed a modest but significant increase across the groups (*p* = 0.048; η^2^ = 0.06). A greater proportion of the participants in the early-AD group also had hypertension, diabetes, and a recent history of falls, with Cramér’s V values indicating moderate effect sizes (*p* < 0.05). There were no significant group differences in sex distribution (*p* = 0.724).

The cognitive and vestibular function scores demonstrated statistically significant group differences, with progressive decline observed from cognitively healthy individuals to those with MCI and early AD (Table 2). Both the MoCA and the MMSE scores were significantly lower in the MCI and AD groups compared to the healthy controls, with all the post-hoc comparisons reaching statistical significance (*p* < 0.01). Similarly, the posturography sway scores increased, and vHIT VOR gain decreased significantly across groups, indicating worsening vestibular function in parallel with cognitive decline. These findings highlight a consistent pattern of deterioration in both cognitive and vestibular domains, with a clear separation between all the diagnostic groups.

Significant group differences were observed for the subjective vestibular symptoms and functional balance performance, with worsening trends from the cognitively healthy individuals to those with MCI and early Alzheimer’s disease (Table 3). The participants with cognitive impairment reported higher DHI scores, indicating greater perceived dizziness-related disability. Objective measures showed a corresponding decline in gait speed and balance time during eyes-closed stance tasks, suggesting impaired postural control and mobility in the cognitively impaired groups. These findings highlight the inter-relationship between vestibular symptoms, balance dysfunction, and cognitive decline in older adults.

**Figure 1:**
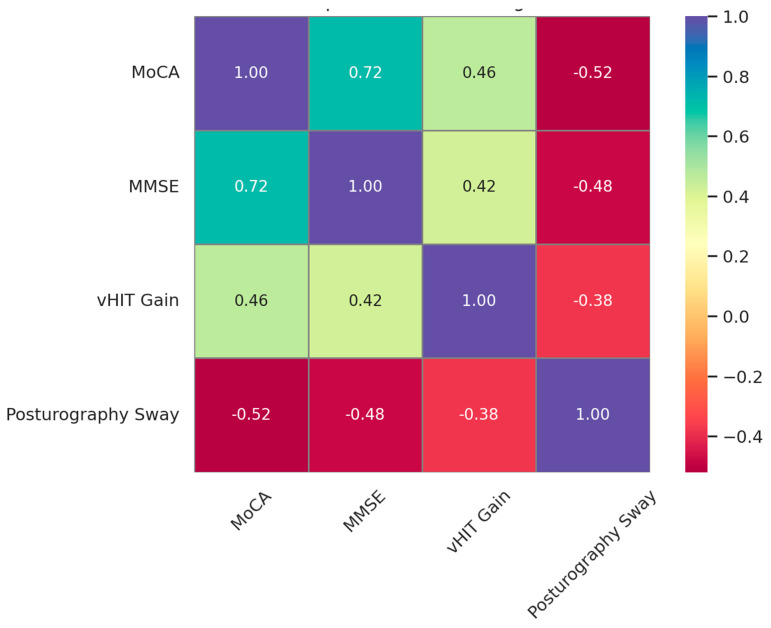
Correlation heatmap showing relationships between vestibular function and cognitive performance measures.

Moderate and statistically significant correlations were observed between the vestibular function measures and the cognitive performance scores, indicating that vestibular integrity is closely associated with cognitive status in older adults (Figure 1). Specifically, greater vHIT gain was positively correlated with the MoCA (*r* = 0.46, *p* = 0.002) and the MMSE scores (*r* = 0.42, *p* = 0.005), while greater posturography sway was negatively correlated with both the MoCA (*r* = −0.52, *p* = 0.001) and the MMSE scores (*r* = −0.48, *p* = 0.003). These findings suggest that reductions in vestibulo-ocular and postural control functions may reflect or contribute to cognitive decline.

The multivariable linear regression analysis identified several independent predictors of cognitive performance, with the models explaining a substantial proportion of the variance in the MoCA (adjusted R^2^ = 0.68) and the MMSE (adjusted R^2^ = 0.65) scores (Table 4). Greater vHIT gain and a faster gait speed were positively associated with both of the cognitive measures, while increased posturography sway and DHI scores were significant negative predictors. The participants’ age and the presence of comorbidities (hypertension/diabetes) were inversely associated with the cognitive scores, whereas higher education was positively associated. All the predictors demonstrated acceptable multicollinearity levels (VIFs < 1.5), supporting the robustness of the regression models. These findings underscore the multidimensional interplay between vestibular function, physical performance, and cognitive health in aging populations.

**Figure 2:**
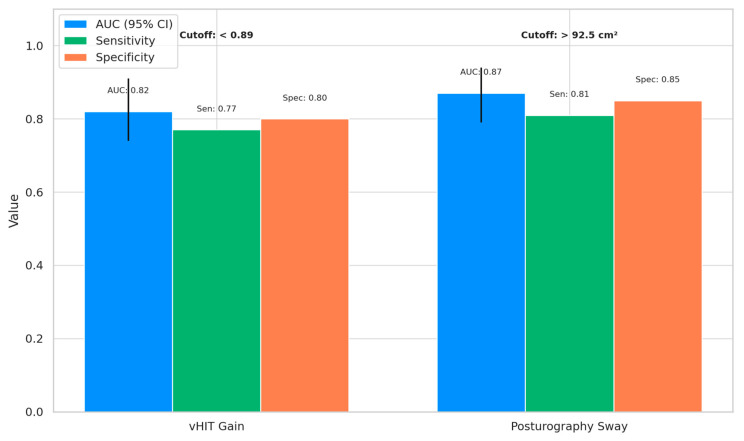
Statistical comparison of ROC metrics for vestibular markers in identifying cognitive impairment.

The receiver operating characteristic (ROC) analysis demonstrated that both vHIT gain and posturography sway were effective in distinguishing the cognitively impaired individuals (MCI and AD) from the healthy controls, with a high diagnostic accuracy (Figure 2). Posturography sway showed the highest discriminative ability, with an AUC of 0.87 (95% CI: 0.79–0.94), sensitivity of 0.81, and specificity of 0.85 at an optimal cutoff of >92.5 cm^2^. Similarly, vHIT gain yielded an AUC of 0.82 (95% CI: 0.74–0.91), with sensitivity and specificity values of 0.77 and 0.80, respectively, at a cutoff of <0.89. These findings highlight the potential utility of vestibular markers in early cognitive screening.

## 4. Discussion

This study aimed to investigate the association between vestibular dysfunction and early cognitive impairment in older adults, with a focus on identifying potential vestibular markers relevant to clinical screening. The findings demonstrated a consistent decline in both cognitive and vestibular function across the spectrum from cognitively healthy individuals to those with mild cognitive impairment and early Alzheimer’s disease. Objective vestibular measures, such as posturography sway and vHIT gain, showed strong associations with cognitive performance and were significant predictors of cognitive status after adjusting for demographic and clinical confounders. Additionally, both vestibular markers exhibited good diagnostic accuracy in distinguishing cognitively impaired individuals from controls. Subjective dizziness severity and balance measures also deteriorated with cognitive decline, further supporting the functional interplay between vestibular integrity and cognition. Collectively, these results reinforce the potential role of vestibular assessments in the early identification of cognitive impairment.

The observed deterioration in both cognitive and vestibular function across diagnostic groups is consistent with accumulating evidence linking central and peripheral vestibular decline to neurodegenerative processes [6]. Previous studies have demonstrated that impairments in the vestibulo-ocular reflex and postural stability are common in individuals with mild cognitive impairment and Alzheimer’s disease, likely due to shared neural substrates involving the hippocampus, cerebellum, and brainstem circuits [26,27]. The current regression findings further support this association, as less vHIT gain and increased posturography sway independently predicted reduced cognitive scores, aligning with the work of Bosmans et al. [9], who reported similar associations between vestibular deficits and memory performance. Additionally, decreased gait speed and an increased dizziness handicap were significantly associated with poorer cognition, corroborating findings by Ikeda et al. [28] and Beck et al. [29], who emphasized the relevance of mobility and balance metrics as early indicators of cognitive decline. The inverse relationship between age, comorbidities, and cognitive function, as well as the positive influence of education, aligns with the established predictors of cognitive reserve and decline [30]. These results, grounded in well-documented neural and functional pathways, underscore the clinical utility of incorporating vestibular and mobility assessments into cognitive screening protocols. Although the regression models statistically controlled for age, the influence of aging on vestibular function is an independent physiological factor that must be considered. Age-related vestibular decline—characterized by the loss of hair cells, reduced vestibulo-ocular reflex gain, and central processing deficits—may affect postural control and eye movement responses [31]. Given that the AD group was significantly older than the control group, part of the observed vestibular dysfunction may be attributable to normal aging processes. Thus, the effects of age and neurodegeneration on vestibular performance may be additive rather than isolated, and future longitudinal studies are needed to disentangle these overlapping influences [32].

The interplay between vestibular dysfunction and specific cognitive domains is supported by shared cortical and subcortical pathways [33]. Both systems rely heavily on the hippocampus and temporoparietal regions, which mediate spatial navigation, visuospatial memory, and attentional control—cognitive areas frequently affected in early Alzheimer’s disease [7]. Vestibular dysfunction has been associated with impairments in mental rotation, wayfinding, and executive control, all of which overlap with early cognitive decline patterns [34]. Although domain-specific testing was not performed in this study, the theoretical convergence highlights the relevance of vestibular markers as potential early indicators of decline beyond global scores alone.

The high diagnostic performance of posturography sway and vHIT gain in identifying cognitive impairment aligns with previous evidence demonstrating vestibular dysfunction as a significant marker of neurodegenerative change [9]. The superior discriminative accuracy of posturography sway is consistent with findings by Dale et al. [35], who noted that impaired postural control reflects early cerebellar and vestibular processing deficits associated with Alzheimer’s pathology. Similarly, the effectiveness of vHIT gain in differentiating cognitive states supports the work of Anson et al. [8], who identified a strong relationship between horizontal VOR deficits and cognitive decline in older adults [8]. The vestibulo-cortical pathways, particularly those involving the temporoparietal junction and the hippocampus, are known to play a role in both spatial orientation and memory function, explaining the convergence of vestibular and cognitive impairment (Smith et al. [10]). Neuroanatomically, the tau pathology characteristic of early Alzheimer’s disease may disrupt vestibular–cognitive pathways by impairing synaptic function in the hippocampus, entorhinal cortex, and retrosplenial cortex—regions that receive vestibular input via multisynaptic projections from the vestibular nuclei through the thalamus [36]. Animal models of vestibular deafferentation demonstrate hippocampal atrophy and disrupted place cell activity, providing strong evidence for a direct link between vestibular input and spatial memory processing [37]. Postmortem studies further support this relationship, showing early tau accumulation in the medial temporal, parietal, and retrosplenial areas—regions known to overlap with vestibular integration pathways [37]. These findings offer a plausible biological framework for the observed association between vestibular dysfunction and early cognitive decline. The diagnostic thresholds identified in this study provide clinically meaningful cutoffs that reinforce the utility of vestibular testing as a complementary approach to traditional cognitive screening [18,38].

The progressive increase in subjective dizziness and the concurrent decline in functional balance measures observed among individuals with MCI and early AD are well-supported by previous research linking vestibular impairment to functional mobility limitations and cognitive dysfunction [39,40]. The higher DHI scores in the cognitively impaired participants reflect the greater severity of their perceived vestibular-related disability, consistent with findings from Anson et al. [8], who reported that self-reported dizziness is significantly associated with poorer balance and cognitive function in aging populations. The decline in gait speed and balance time under eyes-closed conditions is similarly supported by studies showing that cognitive impairment negatively impacts postural control and gait performance due to deficits in sensory integration and attentional resources [19,40]. Moreover, the regression models further confirmed that both DHI scores and gait speed are independent predictors of cognitive performance, aligning with the findings of Pavlou et al. [39], who highlighted mobility measures as sensitive markers of early cognitive decline. These observations emphasize the interplay between vestibular symptoms, sensorimotor integration, and cognitive deterioration in older adults. A further limitation is the partial evaluation of the vestibular system.

This study assessed only horizontal semicircular canal function using vHITs, excluding vertical canal assessments, oVEMP, cVEMP, and caloric testing, which may have led to us underestimating the participants’ multidimensional vestibular impairment. Subjective vestibular symptoms were measured using the DHI, but broader quality-of-life instruments, like SF-12 or SF-36, were not included. Sensory Organization Testing and similar comprehensive vestibular evaluations could enhance future studies. The association between dizziness and cognition may be bidirectional [41]. Vestibular dysfunction can impair spatial and executive functions, while cognitive deficits—particularly in attention, working memory, and emotional regulation—may intensify dizziness perception [42]. Anxiety and cognitive overload may amplify symptoms even without objective vestibular loss, suggesting central perceptual and emotional contributions to subjective dizziness [43]. Additionally, static posturography under eyes-closed conditions reduces visual input but does not isolate vestibular function due to continued somatosensory input. Therefore, increased sway, in this setting, cannot be interpreted as a specific vestibular deficit. The lack of dynamic posturography or sensory conflict paradigms limits the interpretability of our results, and future research should use more targeted postural assessments.

### Limitations and Future Directions

This study has several limitations that must be considered. Its cross-sectional design prevents causal inferences between vestibular dysfunction and cognitive decline; longitudinal studies are necessary to establish temporal relationships. The diagnoses of MCI and early AD were based solely on clinical criteria without neuroimaging or cerebrospinal fluid biomarkers, increasing the risk of misclassification, particularly with the possible inclusion of non-AD dementias, such as vascular cognitive impairment. The sample was limited to a single geographic region in Saudi Arabia, which may affect our results’ generalizability due to cultural, educational, and healthcare system differences. Additionally, recruitment from a vestibular clinic may introduce a referral bias, as the participants may not represent the broader older adult population and could over-represent vestibular abnormalities. While vestibular dysfunction was assessed using horizontal vHITs and posturography, the study did not include vertical canal, otolith organ (e.g., oVEMP, cVEMP), or neuro-vestibular imaging assessments. As a result, multidimensional vestibular impairment may have been underestimated. The vHIT gain threshold used (<0.90) may not align with current normative standards, which are typically <0.80 for horizontal canals. Furthermore, the lack of neuro-orthoptic or oculomotor evaluations means potential central abnormalities—such as impaired smooth pursuit or saccadic dysfunction—may have confounded the vHIT results, despite excluding the participants with visibly unreliable recordings. No comprehensive neuropsychological battery was used, limiting our assessment to global cognitive performance and precluding any analysis of specific cognitive domains. Although the study employed widely accepted tools (MoCA, MMSE) for feasibility, this limits granularity. Lastly, not all studies support the vestibular–cognition association, and discrepancies in findings across the literature suggest that other neural or psychosocial moderators may influence this relationship. Future research should adopt longitudinal, multimodal designs with broader vestibular and cognitive assessments to validate and extend these findings.

## 5. Conclusions

This study demonstrated that vestibular dysfunction, as assessed by posturography sway and vHIT gain, is significantly associated with cognitive impairment in older adults, with clear group-wise differences having been observed across cognitively healthy, MCI, and early-AD populations. Both objective vestibular markers and subjective dizziness symptoms were found to correlate with cognitive performance and independently predicted cognitive scores in multivariable models. Furthermore, posturography sway and vHIT gain showed high diagnostic accuracy in distinguishing cognitively impaired individuals from healthy controls. These findings support the clinical relevance of integrating vestibular assessment into the evaluation of cognitive status in aging populations, particularly for the early identification of mild cognitive impairment and early Alzheimer’s disease. These findings highlight the potential role of objective vestibular assessments—such as vHIT gain and posturography sway—as practical, non-invasive tools for aiding early cognitive screening in geriatric populations.

## Figures and Tables

**Table 1:** Demographic and clinical characteristics of participants.

Variable	Cognitively Healthy (*n* = 30)	MCI (*n* = 30)	Early AD (*n* = 30)	*p*-Value	Effect Size
Age (years)	69.10 ± 4.32	71.30 ± 5.00	73.90 ± 4.85	0.014	η^2^ = 0.09
Education (years)	12.50 ± 2.10	11.20 ± 2.30	9.10 ± 2.00	0.003	η^2^ = 0.12
BMI (kg/m^2^)	26.50 ± 3.20	27.40 ± 3.10	28.10 ± 3.45	0.048	η^2^ = 0.06
Sex (Male/Female)	18/12	16/14	15/15	0.724	Cramér’s V = 0.10
Hypertension (Yes/No)	10/20	16/14	20/10	0.015	Cramér’s V = 0.30
Diabetes (Yes/No)	8/22	12/18	18/12	0.023	Cramér’s V = 0.28
Fall History (Yes/No)	5/25	10/20	16/14	0.012	Cramér’s V = 0.33

BMI: body mass index. MCI: mild cognitive impairment. AD: Alzheimer’s disease. η^2^: Eta Squared. V: Cramér’s V.

**Table 2:** Cognitive and vestibular function scores by group with post-hoc analysis.

Variable	Cognitively Healthy (*n* = 30)	MCI (*n* = 30)	Early AD (*n* = 30)	Overall *p*-Value	Tukey’s Post-Hoc (Healthy vs. MCI)	Tukey’s Post-Hoc (Healthy vs. AD)	Tukey’s Post-Hoc (MCI vs. AD)
MoCA Score (0–30)	27.80 ± 1.30	23.10 ± 1.80	19.40 ± 2.20	0.001	0.004	0.001	0.010
MMSE Score (0–30)	28.50 ± 1.00	26.10 ± 1.70	22.90 ± 2.10	0.002	0.007	0.001	0.013
Posturography Sway Score (cm^2^)	72.30 ± 15.20	104.70 ± 20.30	129.60 ± 25.50	0.001	0.005	0.001	0.008
vHIT VOR Gain (horizontal)	0.96 ± 0.04	0.85 ± 0.06	0.76 ± 0.08	0.001	0.006	0.001	0.009

MoCA: Montreal Cognitive Assessment. MMSE: Mini-Mental State Examination. vHIT: video head impulse test. VOR: vestibulo-ocular reflex. AD: Alzheimer’s disease. MCI: mild cognitive impairment. cm^2^: square centimeters.

**Table 3:** Subjective vestibular symptoms and functional balance by group.

Variable	Cognitively Healthy (*n* = 30)	MCI (*n* = 30)	Early AD (*n* = 30)	*p*-Value
Dizziness Handicap Inventory (DHI) Score (0–100)	10.50 ± 6.30	24.80 ± 9.40	37.90 ± 10.50	0.001
Gait Speed (m/s)	1.24 ± 0.18	1.01 ± 0.20	0.86 ± 0.22	0.003
Balance Test Time (s, eyes-closed single-task)	31.20 ± 5.40	24.30 ± 6.10	18.40 ± 6.50	0.001

DHI: Dizziness Handicap Inventory. m/s: meters per second. AD: Alzheimer’s disease. MCI: mild cognitive impairment. n: number. *p*-value: Probability value.

**Table 4:** Multivariable linear regression: predictors of cognitive scores with model diagnostics.

Outcome	Independent Variable	Beta Coefficient	95% CI	*p*-Value	VIF
MoCA Score	vHIT Gain	3.25	1.40 to 5.10	0.001	1.12
MoCA Score	Posturography Sway	−0.18	−0.29 to −0.07	0.003	1.36
MoCA Score	DHI Score	−0.09	−0.15 to −0.03	0.006	1.18
MoCA Score	Gait Speed	4.12	2.30 to 5.94	0.001	1.44
MoCA Score	Age	−0.26	−0.41 to −0.11	0.002	1.22
MoCA Score	Education	0.41	0.15 to 0.67	0.002	1.41
MoCA Score	Comorbidities (HTN/DM)	−1.15	−2.03 to −0.27	0.012	1.20
MMSE Score	vHIT Gain	2.87	1.25 to 4.49	0.001	1.12
MMSE Score	Posturography Sway	−0.15	−0.27 to −0.03	0.016	1.36
MMSE Score	DHI Score	−0.08	−0.14 to −0.02	0.008	1.18
MMSE Score	Gait Speed	3.78	2.01 to 5.55	0.001	1.44
MMSE Score	Age	−0.23	−0.37 to −0.09	0.003	1.22
MMSE Score	Education	0.39	0.12 to 0.66	0.004	1.41
MMSE Score	Comorbidities (HTN/DM)	−1.02	−1.87 to −0.17	0.021	1.20

MoCA: Montreal Cognitive Assessment. MMSE: Mini-Mental State Examination. vHIT: video head impulse test. DHI: Dizziness Handicap Inventory. HTN: hypertension. DM: diabetes mellitus. CI: confidence interval. VIF: Variance inflation factor. *p*-value: Probability value.

## Data Availability

The raw data supporting the findings of this study are available on Zenodo at https://doi.org/10.5281/zenodo.15550066 (accessed on 24 June 2025). All the versions of the dataset can be cited using this DOI. The dataset includes the anonymized, individual-level data used in the statistical analyses, consistent with the results reported in this manuscript.

## Abbreviations

The following abbreviations are used in this manuscript:

AD	Alzheimer’s disease
ANOVA	analysis of variance
AUC	area under the curve
BMI	body mass index
CI	confidence interval
COP	center of pressure
DHI	Dizziness Handicap Inventory
DM	diabetes mellitus
HTN	hypertension
MCI	mild cognitive impairment
MoCA	Montreal Cognitive Assessment
MMSE	Mini-Mental State Examination
NIA-AA	National Institute on Aging–Alzheimer’s Association
ROC	receiver operating characteristic
VIF	Variance inflation factor
vHIT	video head impulse test
VOR	vestibulo-ocular reflex
